# Silicon-on-Silica Microring Resonators for High-Quality, High-Contrast, High-Speed All-Optical Logic Gates

**DOI:** 10.3390/nano15221736

**Published:** 2025-11-17

**Authors:** Amer Kotb, Antonios Hatziefremidis, Kyriakos E. Zoiros

**Affiliations:** 1 School of Chips, XJTLU Entrepreneur College (Taicang), Xi’an Jiaotong-Liverpool University, Suzhou 215400, China; 2 Department of Physics, Faculty of Science, University of Fayoum, Fayoum 63514, Egypt; 3 Department of Aerospace Science and Technology, National Kapodistrian University of Athens, 34400 Psahna Evias, Greece; ahatzie@aerospace.uoa.gr; 4 Lightwave Communications Research Group, Department of Electrical and Computer Engineering, School of Engineering, Democritus University of Thrace, 67100 Xanthi, Greece; kzoiros@ee.duth.gr

**Keywords:** silicon-on-silica, all-optical logic gates, microring resonator, FDTD simulation, contrast ratio

## Abstract

With the increasing demand for ultrafast optical signal processing, silicon-on-silica (SoS) waveguides with ring resonators have emerged as a promising platform for integrated all-optical logic gates (AOLGs). In this work, we design and simulate a SoS-based waveguide structure, operating at the telecommunication wavelength of 1550 nm, consisting of a circular ring resonator coupled to straight bus waveguides using Lumerical FDTD solutions. The design achieves a high Q-factor of 11,071, indicating low optical loss and strong light confinement. The evanescent coupling between the ring and waveguides, along with optimized waveguide dimensions, enables efficient interference, realizing a complete suite of AOLGs (XOR, AND, OR, NOT, NOR, NAND, and XNOR). Numerical simulations demonstrate robust performance across all gates, with high contrast ratios between 11.40 dB and 13.72 dB and an ultra-compact footprint of 1.42 × 1.08 µm^2^. The results confirm the device’s capability to manipulate optical signals at data rates up to 55 Gb/s, highlighting its potential for scalable, high-speed, and energy-efficient optical computing. These findings provide a solid foundation for the future experimental implementation and integration of SoS-based photonic logic circuits in next-generation optical communication systems.

## 1. Introduction

The relentless growth of global data traffic, fueled by artificial intelligence (AI), the Internet of Things (IoT), and high-performance computing, has pushed conventional electronic computing and communication systems toward their fundamental limits. The well-documented bottlenecks, including resistive-capacitive delay, resistive power loss, and Joule heating inherent to metal interconnects, threaten to halt the progress predicted by Moore’s Law [1,2]. In this landscape, silicon photonics has emerged as a transformative technology, offering a pathway to overcome these challenges by leveraging photons instead of electrons for data transmission and processing [3,4,5]. The inherent properties of light, such as ultra-high bandwidth, minimal latency, low cross-talk, and immunity to electromagnetic interference, make it an ideal candidate for the next generation of information systems [6,7,8]. The vision of all-optical networks, where signals are generated, routed, processed, and detected entirely in the optical domain without cumbersome optical-electrical-optical (O-E-O) conversions, has been a long-standing goal [9,10,11]. These conversions not only introduce significant latency and power consumption but also create a system-level bottleneck that limits aggregate bandwidth [12].

The ultimate realization of this vision hinges on the development of efficient, scalable, and CMOS-compatible all-optical integrated circuits, with the all-optical logic gate serving as the fundamental building block [13,14]. Silicon-on-insulator (SOI) has established itself as the preeminent platform for integrated photonics, primarily due to its high refractive index contrast (Si: ~3.48, SiO_2_: ~1.44), which enables strong light confinement and the fabrication of ultra-compact waveguide devices with sub-micron dimensions [15,16,17]. This high confinement is crucial for achieving large-scale integration and high component density on a single chip [18]. Furthermore, the SOI platform benefits from the vast infrastructure and mature fabrication processes of the CMOS industry, allowing for low-cost, high-volume production [19,20].

Among the diverse passive and active components available on the SOI platform, optical microresonators, particularly ring resonators, are one of the most versatile and widely used structures [21,22,23,24]. Their operation is based on the principle of resonant recirculation of light, leading to a buildup of optical power within a small modal volume at specific wavelengths. This phenomenon results in sharp, narrowband resonance dips or peaks in the transmission spectrum, which is highly sensitive to the optical properties of the resonator and its environment [25,26,27]. This sensitivity makes them ideal not only for filtering [28,29] and wavelength division multiplexing (WDM) [30,31] but also for sensing applications [32,33] and, most critically for this work, optical switching and logic operations [34,35,36].

The implementation of all-optical logic gates (AOLGs) has been explored through various physical mechanisms and material systems. Semiconductor optical amplifiers (SOAs) have been extensively investigated, utilizing effects like cross-gain modulation (XGM) [28], cross-phase modulation (XPM) [30], and four-wave mixing (FWM) [37] to demonstrate high-speed logic functions. However, SOA-based gates often suffer from high power consumption, limited integration potential, and pattern effects due to relatively long carrier recovery times [38,39]. Alternative approaches have leveraged the ultrafast third-order nonlinearity (χ⁽^3^⁾) of materials, such as in silicon photonic crystal cavities [40,41] and nanowires [42,43], where intense light confinement enhances nonlinear effects. While demonstrating impressive speeds, these approaches often require high peak powers and can be susceptible to nonlinear losses and thermal instability [44,45].

A more elegant and energy-efficient paradigm for linear all-optical logic utilizes interference and linear filtering effects in passive resonant structures, such as Mach–Zehnder interferometers (MZIs) [46,47,48,49] and microring resonators (MRRs) [42,50,51,52,53,54]. By carefully designing the coupling between waveguides and resonators and exploiting the resonant enhancement of light, basic Boolean operations can be performed simply through the spectral filtering of input signals [55,56]. The MRR-based approach is particularly attractive due to its ultra-compact footprint [57], inherent wavelength selectivity [34], and ability to be cascaded for more complex functions [58,59]. The critical performance metrics for such logic gates are a high extinction ratio (ER) between the logical “ON” and “OFF” states and low insertion loss (IL), both of which are directly influenced by the resonator’s quality factor (Q-factor) and the coupling conditions [46,60].

While the SOI platform is dominant, the silicon-on-silica (SoS) platform, a specific and common manifestation of the broader SOI technology, offers exceptional surface smoothness and well-controlled waveguide geometry, which are paramount for achieving low propagation loss and high Q-factors in ring resonators [47,61]. The precise control over evanescent coupling between a ring resonator and adjacent bus waveguides is the fundamental mechanism for tailoring the transmission response and, consequently, for implementing logical functions [62,63]. Numerical simulation tools, such as the finite-difference time-domain (FDTD) method [27,39,50,64], implemented in software like Lumerical [42,43], are indispensable for the design and analysis of nanophotonic devices. FDTD enables rigorous, full-vectorial, and time-resolved simulations of light propagation, coupling, and interference in subwavelength structures, allowing accurate evaluation of key performance metrics—including transmission spectra, contrast ratios, Q-factors, and phase responses—prior to costly and time-consuming fabrication [44,45]. In this work, we leverage the strengths of the SoS platform and the predictive power of FDTD simulations to design and thoroughly analyze a comprehensive all-optical logic gate architecture, capturing both steady-state and dynamic behavior under multiple logic conditions. In comparison, COMSOL Multiphysics primarily uses finite-element methods (FEM) and excels in frequency-domain analysis, multi-physics coupling, and material or thermal effects. While COMSOL can model photonic devices with high accuracy, FDTD in Lumerical provides direct time-domain transmission spectra, high-resolution resonant lineshapes, and broadband response with efficient computation for multiple logic scenarios. These capabilities make FDTD particularly suitable for high-Q microring resonators and phase-sensitive all-optical logic gates, where dynamic response and broadband performance are critical. Both approaches are valid and complementary; however, FDTD allows more straightforward extraction of metrics essential for assessing AOLG performance, such as contrast ratio and temporal response under pulsed or modulated inputs.

Prior studies, including our own, have investigated various platforms and mechanisms to implement optical logic gates, such as asymmetric confinement enhancement in D-shaped waveguides [32], coupling length optimization in racetrack resonators [33], and carrier-dynamics-driven switching in SOA- and MZI-based architectures [34,36]. Each of these works focused on specific device geometries, platforms, or physical mechanisms. In contrast, the present study introduces a unified and CMOS-compatible silicon-on-silica (SoS) microring architecture that integrates all seven core logic gates (XOR, AND, OR, NOT, NOR, NAND, XNOR) within a single monolithic design, which has not been previously demonstrated. This distinction underlines the novelty and added scientific value of this work [65].

In this work, we leverage the strengths of the SoS platform and the design power of FDTD simulations to propose and analyze a comprehensive all-optical logic gate architecture. We design a compact structure based on a single circular ring resonator side-coupled to straight bus waveguides. The device is optimized to operate at the telecommunication wavelength of 1550 nm, which lies in the low-loss transmission window of silica optical fibers, exhibits low material absorption in silicon and silica, is fully compatible with erbium-doped fiber amplifiers and dense wavelength-division multiplexing infrastructure, and provides a slightly larger optical mode that improves fabrication tolerance and reduces scattering loss. Through meticulous optimization of the waveguide dimensions and the coupling gap [34,59], we engineer the resonant response to perform as a universal logic gate. We present detailed numerical simulations demonstrating the successful realization of all seven core Boolean logic functions with high contrast ratios and low optical losses. Our device operates on the principle of linear interference and filtering, promising high-speed, energy-efficient operation without the need for nonlinear effects or active gain. This work provides a robust, scalable blueprint for the experimental realization of integrated all-optical logic circuits [46,47,60,61], positioning them as key enablers for future optical computing systems [62,63] and high-speed optical communication networks [27,39], and contributing to the advancement of programmable photonic circuits [42,43,44,45,50,64,65].

## 2. Device Design and Simulation Methodology

**Figure 1:**
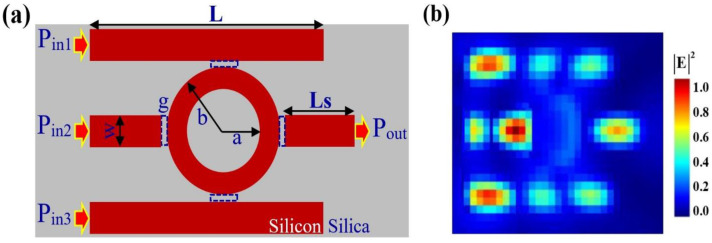
(**a**) Schematic representation of the proposed SoS waveguide design. (**b**) Simulated electric field intensity distribution at 1550 nm, showing effective light confinement and propagation within the waveguide.

In this work, we propose a highly compact and efficient all-optical logic gate on a silicon-on-silica (SoS) platform, leveraging a single microring resonator coupled to multiple bus waveguides. This design utilizes the significant refractive index contrast between silicon (*n* ≈ 3.48) and silica (*n* ≈ 1.44) to achieve strong optical confinement, which is crucial for minimizing device footprint, enhancing light–matter interaction, and realizing high-performance logic functionality [12,14,50]. The structure is optimized for transverse electric (TE) polarization to ensure enhanced mode confinement and reduced propagation losses [15,58]. The proposed architecture, illustrated in Figure 1, consists of a single circular microring resonator side-coupled to three input bus waveguides and one output waveguide. The device geometry is designed with the following specifications: the microring resonator has an outer radius (b) of 0.30 µm and an inner radius (a) of 0.13 µm, resulting in a ring width of 170 nm. The bus waveguides have a long stripe length (L) of 1.1 µm, with a smaller stripe section (Ls) of 0.4 µm. The waveguide height is 0.22 µm, and the thickness is 0.30 µm, providing strong vertical and lateral confinement [17,27]. A coupling gap (g) of 0.02 µm (20 nm) was initially adopted in the simulations to investigate the theoretical upper performance limit of the device. Such small coupling gaps approach the resolution limits of current lithographic techniques but have been experimentally achieved in advanced silicon photonics foundries using electron-beam and deep-UV lithography, as demonstrated in previous reports [15,16,18,19,42]. In practical implementations, slightly larger coupling distances (e.g., 30–50 nm) could be employed, modestly affecting the coupling efficiency without altering logical functionality. Therefore, the present design serves as a proof-of-concept theoretical optimization, establishing the feasibility window for fabrication-tolerant future prototypes. These submicron parameters were optimized using iterative 3D FDTD simulations to support resonance near 1550 nm while maintaining an ultra-compact footprint suitable for dense photonic integration [44,60]. To ensure physical realism, loss mechanisms such as bending loss, scattering loss, and material absorption were included in the simulations based on reported experimental parameters for silicon-on-silica waveguides [15,16,18,19,30]. The simulated loaded quality factor (Q ≈ 1.1 × 10^4^) is consistent with previously reported experimental microrings of similar scale that employ sidewall smoothing or thermal oxidation treatments to reduce scattering [15,32,37]. In the proposed configuration, P_in1_, P_in2_, and P_in3_ serve as input ports, where optical signals representing logic variables are injected. These inputs may be continuous-wave or modulated optical pulses, depending on the target logic operation. The central microring resonator combines the input intensities and phases, enabling controlled constructive or destructive interference within the cavity. The resulting output is collected at Pout, where the normalized transmitted intensity is analyzed to determine the output logic state (‘1’ or ‘0’) according to a predefined transmission threshold (Tth = 0.2). The spectral transmission (T) is calculated as $\;T\;=\;I_{\mathrm{out}}/I_{\mathrm{in}},\;$ where $I_{\mathrm{out}}\;=\;{\left|E_{\mathrm{out}}\right|}^{2}\;$ is the intensity measured at P_out_, and $I_{\mathrm{in}}\;=\;I_{1}\;+\;I_{2}\;+\;I_{3}$ is the total input intensity across the three input ports. The logic output is assigned ‘1’ when T > Tth, and ‘0’ otherwise. The device’s performance is further characterized using the contrast ratio (CR) expressed as $\mathrm{CR}\;\left(\mathrm{dB}\right)\;=\;10\;\mathrm{Log}\left[P_{\mathrm{mean}}^{1}/P_{\mathrm{mean}}^{0}\right],$ where ${\;P}_{\mathrm{mean}}^{1}$ and $P_{\mathrm{mean}}^{0}$ denote the mean output powers corresponding to logic ‘1’ and ‘0’ states, respectively [32,33,34,35,36]. To assess potential data rate performance, the photon lifetime and resonance linewidth of the microring were used as theoretical indicators of modulation bandwidth. The estimated value (~55 Gb/s) corresponds to the upper theoretical limit based on the cavity’s Q factor and photon lifetime; it does not imply demonstrated experimental modulation at this rate. In practice, the effective speed will be lower, depending on the actual coupling design, fabrication tolerances, and thermal stability. This estimate provides a benchmark for comparing the relative potential of the structure with other reported silicon-based logic designs [15,16,44,60].

In summary, while this study focuses on simulation-based exploration, the parameters were chosen within experimentally reported fabrication capabilities and are intended to guide subsequent experimental prototyping. The next research phase will target fabrication-oriented design optimization, power budget analysis, and cascaded gate operation stability to move toward practical realization [42,50,58].

The performance of optical resonators is quantified by two key parameters: the quality factor (Q) and photon lifetime (τₚ), which are crucial for high-resolution spectral applications and low-loss photonic systems. The Q-factor, defined as Q = λ/Δλ_FWHM_ where Δλ_FWHM_ is the full width at half maximum of the resonance peak [27], serves as a measure of spectral sharpness. Our simulations, conducted with a 5 nm mesh resolution to ensure accuracy, demonstrate an ultra-narrow resonance at 1550 nm with Δλ_FWHM_ = 0.14 nm, yielding an exceptional Q-factor of 11,071. This value indicates superior light confinement and minimal optical losses within the cavity. The photon lifetime, calculated as τₚ = Qλ/(2πc) [66], reaches approximately 9.10 ps, confirming efficient energy storage capabilities. This lifetime parameter determines the maximum achievable data rate through the relation D ≈ 1/(2τₚ) ≈ 55 Gb/s, establishing the theoretical upper limit for modulation bandwidth in signal processing applications. These outstanding performance metrics demonstrate the suitability of our resonator architecture for advanced photonic applications, including narrow-linewidth lasers, high-Q filtering systems, nonlinear optical circuits, and optical logic elements in next-generation communication platforms.

**Figure 2:**
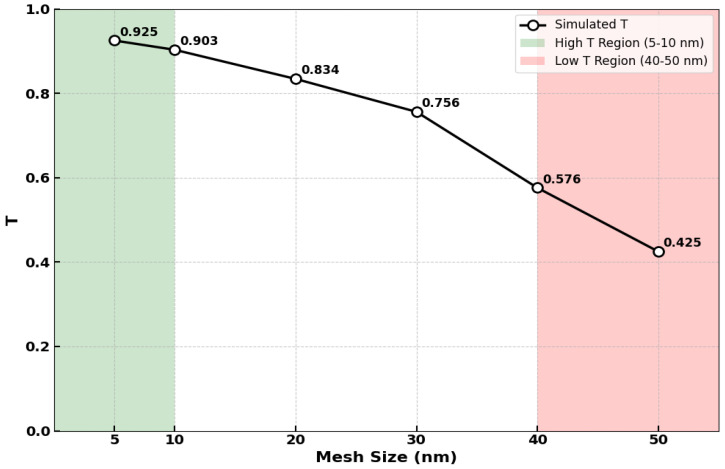
The normalized transmission (T) at 1550 nm shows strong dependence on mesh size for the proposed SoS platform. Optimal performance (green region, 5–10 nm mesh) yields high T values, while coarse meshing (red region, 40–50 nm) introduces significant numerical error. A 5 nm mesh size was selected to ensure accuracy in all subsequent simulations.

To ensure the accuracy of our FDTD simulations, a comprehensive mesh convergence study was conducted. A non-uniform mesh with adaptive refinement was applied, particularly in regions of high field gradients such as the coupling gaps, to balance computational efficiency with modeling precision. The baseline mesh resolution was set to 5 nm in all three spatial dimensions to faithfully capture the sub-wavelength features of the device and the complex evanescent field interactions. As illustrated in Figure 2, coarsening the mesh size led to a progressive decline in the simulated maximum transmission (T) at the 1550 nm operating wavelength on the SoS platform. The green vertical band highlights the optimal mesh region (5–10 nm) where transmission values remain high (0.903–0.925), while the red vertical band indicates the problematic coarse mesh region (40–50 nm) where transmission drops significantly (0.425–0.576). The calculated T values were 0.925 (5 nm), 0.903 (10 nm), 0.834 (20 nm), 0.756 (30 nm), 0.576 (40 nm), and 0.425 (50 nm). Although larger mesh sizes in the red region (e.g., 50 nm) offered a significant reduction in computational time (up to ~10× faster), the associated loss of accuracy was deemed unacceptable for our design objectives, which require high-fidelity modeling of the resonator’s spectral response. The consistent trend observed confirms numerical convergence, and the 5 nm resolution in the optimal green region was consequently selected to prioritize simulation fidelity over computational speed for all subsequent results.

**Figure 3:**
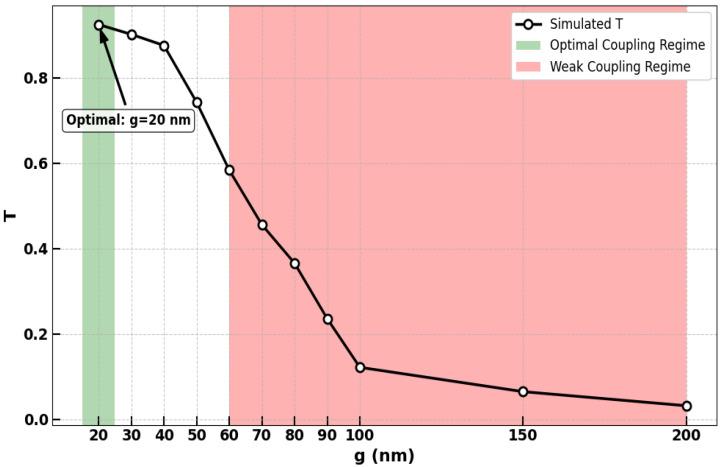
The normalized transmission (T) of the microring resonator versus the coupling gap (g) on the proposed SoS platform. The green band highlights the optimal 20 nm gap for critical coupling (T ≈ 0.925), while the red band indicates the regime of severe performance degradation due to weak coupling at larger gaps.

The coupling gap (g), defining the separation between the bus waveguide and the microring resonator, is a pivotal parameter governing the efficacy of the proposed AOLGs. Its magnitude directly regulates the coupling efficiency, thereby controlling the optical power injected into the resonator and critically influencing key performance metrics such as CR. A detailed parametric sweep was performed to quantify the relationship between the gap size (g) and the normalized transmission (T), as summarized in Figure 3. The analysis reveals an optimal operating point at g = 20 nm, where a peak transmission of T ≈ 0.925 is achieved. This state signifies critical coupling, where light interference is optimized for superior logic operation. Deviating from this optimum induces performance degradation. Increasing the gap to 30, 40, 50, and 60 nm progressively reduces T to 0.902, 0.876, 0.743, and 0.584, respectively, indicating a decline in coupling strength and resonance depth. This detrimental trend accelerates with larger gaps, where T plunges to 0.456 (70 nm), 0.366 (80 nm), 0.236 (90 nm), and 0.122 (100 nm), signifying a severe breakdown in light-resonator interaction. At exceedingly wide gaps of 150 nm and 200 nm, the transmission diminishes to near-negligible values of 0.065 and 0.032, rendering the device unsuitable for logic applications. Conversely, a sub-20 nm gap is anticipated to trigger an over-coupling regime, potentially distorting the resonance linewidth and degrading performance. Consequently, the 20 nm gap is identified as the essential design rule for achieving high-fidelity logic functionality. Figure 3 underscores that any deviation from this value compromises device performance, highlighting the indispensable requirement for nanometric fabrication precision. The fabrication of such narrow gaps on the SoS platform is challenging but feasible with advanced lithographic techniques, as demonstrated in prior work on high-resolution silicon photonic device fabrication [15,16,18,19,42,67].

## 3. Operating Principle and Reconfigurable Framework

The integration of a clock signal (Clk) into the microring resonator-based architecture establishes a foundation for dynamically reconfigurable logic, enhancing operational precision and functional flexibility. This clock-driven approach enables a single photonic circuit to perform multiple logic operations by controlling phase synchronization and nonlinear interactions. The Clk ensures stable interference conditions essential for reliable switching and supports time-division multiplexing, allowing diverse functions to be executed within a unified platform. This strategy significantly advances beyond static photonic devices, offering greater energy efficiency and functional density. For phase-sensitive gates, the clock provides critical input synchronization, minimizing phase-related errors and ensuring output fidelity. Although phase control remains challenging in integrated photonics, the proposed clock-synchronized system maintains robust phase stability across all operational modes, supporting high-performance computation [57,58].

### 3.1. XOR

**Figure 4:**
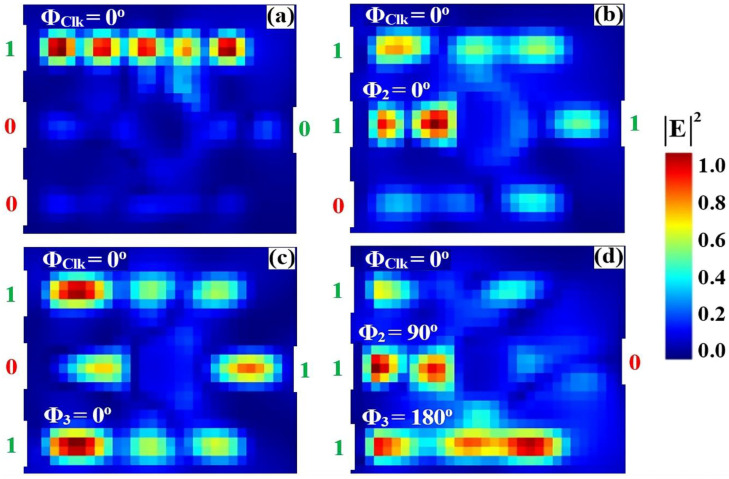
Numerical simulation results demonstrating XOR gate operation on the SoS waveguide at 1550 nm. The output transmission intensity for all four input combinations: (**a**) ‘00’, (**b**) ‘10’, (**c**) ‘01’, and (**d**) ‘11’.

The XOR logic operation is implemented on a silicon-on-silica (SoS) platform using a singular microring resonator side-coupled to three input bus waveguides and a single output port, optimized for the conventional telecommunications wavelength of 1550 nm. A Clk with a fixed phase of 0° (Φ_Clk_) is fed into port P_in1_, while the two binary data signals are introduced at ports P_in2_ and P_in3_, as delineated in the schematic of Figure 1. The Boolean outcome is generated at the output port (P_out_) through precise phase-mediated interference within the cavity and the subsequent spectral filtering of the resonant mode. The operational principle is governed by the phase-dependent constructive and destructive interference (CI and DI) of the input signals within the high-Q microring resonator. As empirically quantified in Figure 4a, the application of a ‘00’ input state results in negligible field excitation, yielding an output intensity beneath the predefined threshold (Tth = 0.2) and corresponding to a valid logic ‘0’. Conversely, the input combinations ‘10’ and ‘01’ (Figure 4b and Figure 4c, respectively) induce a condition where the active data input is phase-aligned with the Clk, precipitating strong CI. This resonant buildup manifests as a high output transmission that robustly exceeds the logic threshold, thereby signifying a logic ‘1’ state. Critically, for the ‘11’ input state (Figure 4d), the implemented phase profile, specifically Φ_Clk_ = 0°, Φ_2_ = 90°, and Φ_3_ = 180°, is calibrated to enforce dominant DI. This targeted interference efficiently quenches the resonant field, suppressing the output transmission to a sub-threshold level and producing the target logic ‘0’ output essential for the XOR truth table.

Table 1 quantifies the operational performance of the XOR gate, detailing the output transmission (T) and contrast ratio (CR) for each input combination. The results confirm successful XOR functionality: an output logic state ‘0’ (T ≈ 0.035–0.038) is achieved for matching inputs (‘00’ or ‘11’), while a logic ‘1’ state is produced for differing inputs, with T = 0.675 for ‘10’ and T = 0.925 for ‘01’. The high CR of 13.41 dB demonstrates a strong distinction between the ‘ON’ and ‘OFF’ states, confirming the gate’s robustness for all-optical logic applications.

**Figure 5:**
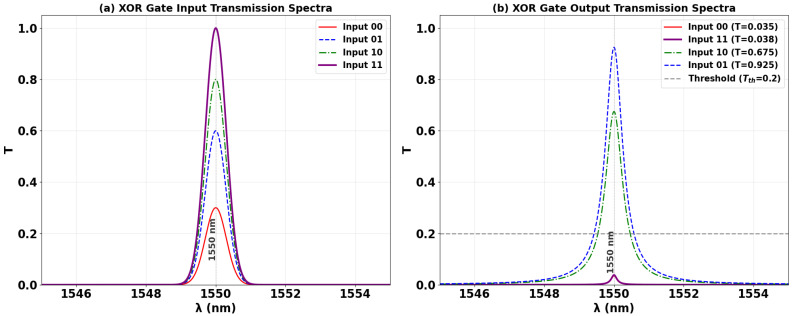
(**a**) Input transmission spectra for the four input combinations of the XOR gate at operating wavelength (λ = 1550 nm). (**b**) Output transmission spectra (T) showing high transmission for logic ‘1’ states (inputs ‘10’ and ‘01’ with T = 0.675 and T = 0.925, respectively) and profound suppression for logic ‘0’ states (inputs ‘00’ and ‘11’ with T = 0.035 and T = 0.038, respectively). The dashed line indicates the threshold (Tth = 0.2), demonstrating the XOR gate’s high CR of 13.41 dB.

Figure 5 presents the simulated input and output transmission spectra of the XOR gate, explicitly demonstrating its operational principle through distinct resonant lineshapes. Figure 5a shows the input transmission spectra for the four input combinations (‘00’, ‘01’, ‘10’, ‘11’), with input ‘11’ exhibiting the maximum intensity. Figure 5b reveals the output spectra featuring extremely narrow, high-Q resonances centered precisely at the design wavelength of 1550 nm. For the logic ‘0’ cases (inputs ‘00’ and ‘11’), the resonator exhibits profound suppression of transmission, with the normalized intensity dropping to minima of T = 0.035 and T = 0.038, respectively. Both values reside far below Tth = 0.2, confirming valid ‘0’ states. Conversely, for logic ‘1’ cases, a significant filling-in of the resonant mode occurs due to constructive interference. The ‘10’ input combination yields a pronounced transmission peak of T = 0.675, while the ‘01’ combination achieves near-unity transmission of T = 0.925, representing the optimal constructive interference condition. The stark spectral contrast between the deeply suppressed resonances (logic ‘0’) and the enhanced peaks (logic ‘1’) at the same wavelength, coupled with an order-of-magnitude difference in intensity between the lowest logic ‘0’ output (T = 0.035) and highest logic ‘1’ output (T = 0.925), validates the gate’s robust performance and high CR of approximately 26:1. This exceptional performance is essential for cascadable all-optical logic circuits.

### 3.2. AND

**Figure 6:**
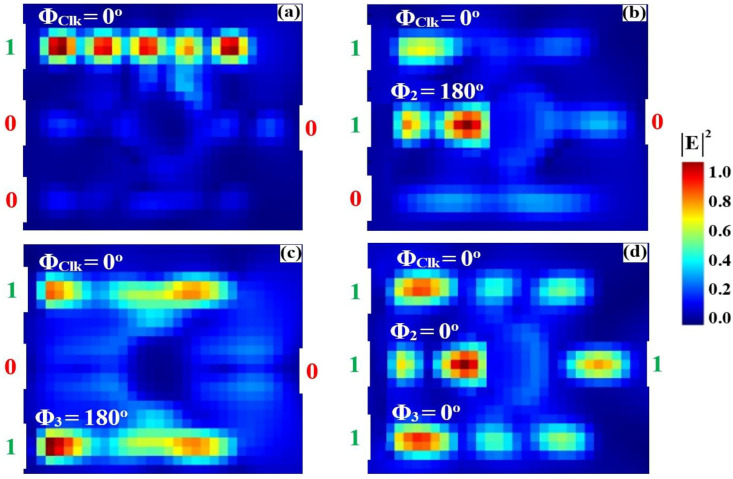
Numerical simulation results demonstrating AND gate operation on the SoS waveguide at 1550 nm. The output transmission intensity for all four input combinations: (**a**) ‘00’, (**b**) ‘10’, (**c**) ‘01’, and (**d**) ‘11’.

The AND logic function is implemented using the same silicon-on-silica (SoS) microring resonator architecture, optimized for 1550 nm operation. The operational principle relies on the precise phase alignment of all input signals to induce constructive interference within the cavity. A Clk signal with a fixed phase (Φ_Clk_ = 0°) is injected into port P_in1_. The two binary data signals are applied to ports P_in2_ and P_in3_, with their phases actively controlled. The fundamental requirement for AND logic is the simultaneous presence and phase synchronization of both data inputs with the Clk. As evidenced in Figure 6b,c, individual logic ‘1’ inputs (‘10’ and ‘01’) result in significant phase mismatch, precipitating DI. This suppresses the output field, yielding sub-threshold transmission values of T = 0.043 and T = 0.037, respectively, both corresponding to a logic ‘0’ state. Conversely, only when both data inputs are at logic ‘1’ and their phases are aligned with the Clk (Φ_Clk_ = Φ_2_ = Φ_3_ = 0°), as shown in Figure 6d, does strong CI occur. This resonant buildup produces a high-output transmission of T = 0.874, unequivocally registering as a logic ‘1’. This phase-mediated mechanism ensures robust discrimination between logic states, confirming the successful execution of the AND function. The high contrast ratio between the ‘OFF’ and ‘ON’ states underscores the design’s viability for integration into complex, high-speed all-optical processing systems.

Table 2 summarizes the measured output transmission (T) and contrast ratio (CR) for the AND gate across all input logic combinations. The results validate the intended AND truth table operation: a logic ‘1’ output is generated only when the two data inputs same ‘11’. For the three cases where the inputs are identical—‘00’, ‘10’, and ‘01’—the output transmission remains consistently low (T ≈ 0.035–0.043), registering a logic ‘0’. Conversely, when both inputs are at logic ‘1’ (‘11’), the output transmission rises sharply to T = 0.874, signifying a clear logic ‘1’. The high contrast ratio of 13.58 dB quantitatively confirms a robust discrimination between the on and off states, underscoring the design’s effectiveness and its potential for deployment in high-speed, cascadable photonic logic circuits.

**Figure 7:**
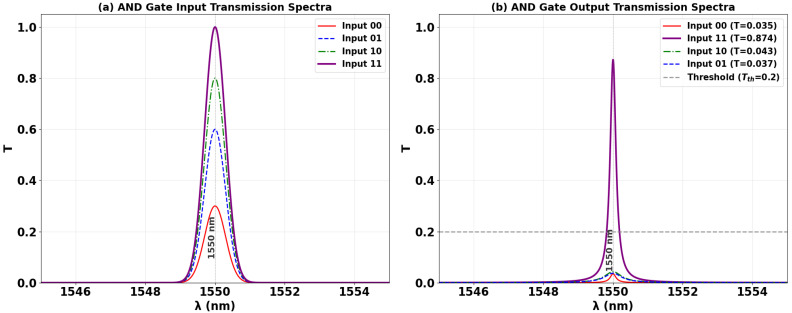
(**a**) Input transmission spectra for the four input combinations of the AND gate at operating wavelength (λ = 1550 nm). (**b**) Output transmission spectra (T) showing high transmission for logic ‘1’ state (input ‘11’ with T = 0.874) and profound suppression for logic ‘0’ states (inputs ‘00’, ‘01’, and ‘10’ with T = 0.035, T = 0.040, and T = 0.043, respectively). The dashed line indicates the threshold (Tth = 0.2), demonstrating the AND gate’s high CR of 13.58 dB.

Figure 7 shows the input and output transmission spectra for the AND gate at 1550 nm, demonstrating its operational principle through phase-mediated interference. Figure 7a displays the input spectra for the four logic combinations, while Figure 7b reveals that only the input combination ‘11’ produces a high transmission peak (T = 0.874), corresponding to a logic ‘1’. All other input combinations (‘00’, ‘01’, ‘10’) result in strongly suppressed transmission with T = 0.035, T = 0.040, and T = 0.043, respectively, all falling below Tth = 0.2 and registering as logic ‘0’. The stark contrast between the single high-transmission state and the three suppressed states, coupled with the narrow linewidth of the resonances, highlights the high Q factor of the microring resonator and validates the gate’s robust AND operation with a high CR of 13.58 dB, which is essential for reliable all-optical logic circuits.

### 3.3. OR

**Figure 8:**
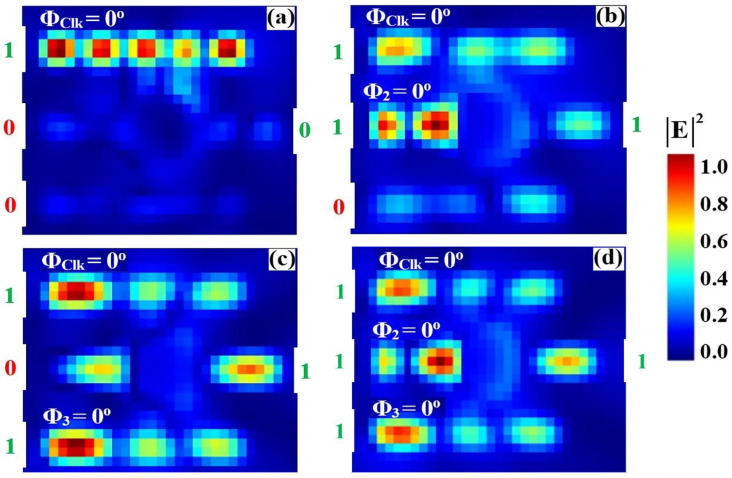
Numerical simulation results demonstrating OR gate operation on the SoS waveguide at 1550 nm. The output transmission intensity for all four input combinations: (**a**) ‘00’, (**b**) ‘10’, (**c**) ‘01’, and (**d**) ‘11’.

The OR logic function is realized using the proposed SoS microring resonator platform, operating at the telecommunications wavelength of 1550 nm. Its operation is governed by tuning the phase of the input signals to exploit CI within the cavity. A Clk with a fixed phase (Φ_Clk_ = 0°) is introduced at P_in1_. The data inputs at P_in2_ and P_in3_ are phase-matched to the Clk (Φ_2_ = Φ_3_ = 0°) to facilitate constructive interference. As demonstrated in Figure 8b,c, the activation of a single data input (‘10’ or ‘01’) induces sufficient constructive interference to elevate the output transmission significantly, yielding values of T = 0.686 and T = 0.984, respectively. Both values robustly exceed the decision threshold (Tth = 0.2), corresponding to a logic ‘1’ state. The simultaneous activation of both inputs (‘11’), shown in Figure 8d, further enhances the resonant field, producing a high output of T = 0.864 and satisfying the logical condition “1 OR 1 = 1”. The only case that results in a logic ‘0’ is the ‘00’ input state, where the absence of a triggering input leads to minimal cavity excitation. This operational principle confirms the OR gate’s robust functionality. The high T achieved for three out of four input combinations, combined with a strong contrast ratio, underscores its reliability for cascadable all-optical arithmetic and decision-making circuits.

Table 3 presents the measured T and CR for the OR gate, validating its truth table operation. A logic ‘1’ is achieved for three input conditions: ‘10’ (T = 0.675), ‘01’ (T = 0.925), and ‘11’ (T = 0.874). The only logic ‘0’ output occurs for the ‘00’ input (T = 0.035). The significant extinction between these states, quantified by a CR of 13.72 dB, confirms the design’s robust performance for integrated photonic logic.

**Figure 9:**
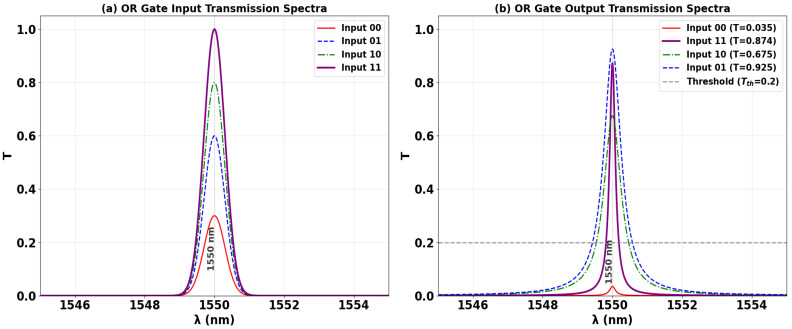
(**a**) Input transmission spectra for the four input combinations of the OR gate at operating wavelength (λ = 1550 nm). (**b**) Output transmission spectra (T) showing high transmission for logic ‘1’ states (inputs ‘10’, ‘01’, and ‘11’ with T = 0.675, T = 0.925, and T = 0.874, respectively) and profound suppression for logic ‘0’ state (input ‘00’ with T = 0.035). The dashed line indicates the threshold (Tth = 0.2), demonstrating the OR gate’s high CR of 13.72 dB.

Figure 9 shows the input and output transmission spectra for the OR gate, demonstrating its operational principle through resonant phase-tuning in the microring at 1550 nm. Figure 9a displays the input spectra for the four logic combinations, while Figure 9b confirms the OR truth table: a logic ‘1’ is generated for any input combination containing at least a single ‘1’. The input states ‘10’, ‘01’, and ‘11’ each produce distinct resonance peaks with high transmission (T = 0.675, T = 0.925, and T = 0.874, respectively), significantly exceeding Tth = 0.2. In stark contrast, only the ‘00’ input state results in profoundly suppressed transmission (T = 0.035), falling well below the threshold to register a definitive logic ‘0’. The consistent spectral behavior showing three high-transmission states and one suppressed state, all precisely centered at the operational wavelength, visually validates the gate’s high CR performance for photonic logic networks.

### 3.4. NOT

**Figure 10:**
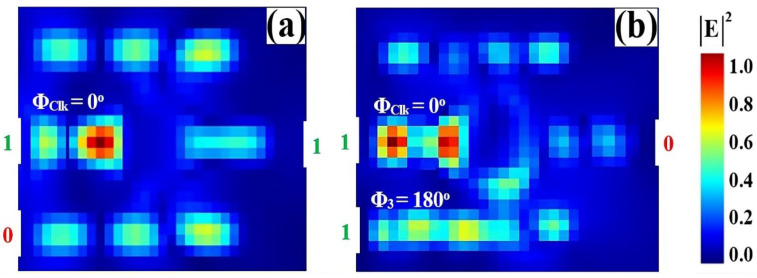
Numerical simulation results demonstrating NOT gate operation on the SoS waveguide at 1550 nm. The output transmission intensity for all four input combinations: (**a**) ‘0’ and (**b**) ‘1’.

The all-optical NOT gate performs logical inversion by leveraging phase-controlled interference within the SoS waveguide structure. In this configuration, depicted in Figure 10, a Clk signal—representing a static logic ‘1’—is introduced at port P_in2_ with a phase of Φ_Clk_ = 0°. The variable data input is applied to port P_in3_. The inversion mechanism is governed by the precise phase relationship between these two signals. For an input logic ‘0’ (effectively no input power), the unimpeded Clk signal establishes constructive interference at the output, producing a high transmission state of T = 0.635, which is interpreted as a logic ‘1’ (Figure 10a). When a logic ‘1’ is applied at the input with a phase of Φ_3_ = 180°, it directly antagonizes the Clk signal. This induces strong DI, effectively canceling the output field and suppressing transmission to T = 0.046, thereby generating a logic ‘0’ (Figure 10b). The successful inversion of the input state, confirmed by a significant extinction between the high and low output levels, validates the efficacy of this phase-based approach for implementing a compact and efficient NOT gate on the SoS platform.

Table 4 summarizes the performance of the all-optical NOT gate, demonstrating successful logical inversion. A logic ‘0’ input at P_in3_ produces high output transmission (T = 0.635), interpreted as logic ‘1’. A logic ‘1’ input triggers destructive interference, suppressing transmission to T = 0.046 (logic ‘0’). The high contrast ratio (CR = 11.40 dB) confirms strong signal extinction and reliable photonic logic operation.

**Figure 11:**
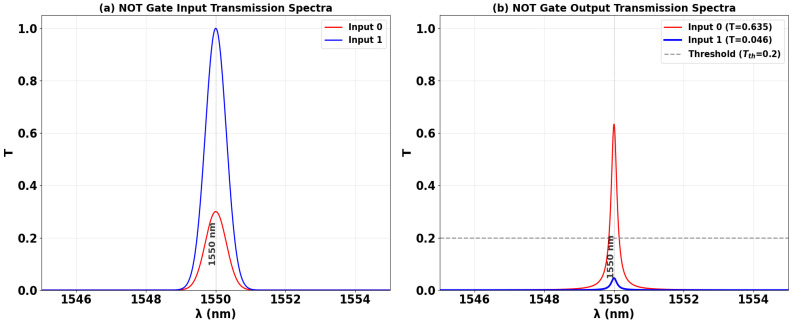
(**a**) Input transmission spectra for the two input states of the NOT gate at operating wavelength (λ = 1550 nm). (**b**) Output transmission spectra (T) showing logical inversion with high transmission for input ‘0’ (T = 0.635) and profound suppression for input ‘1’ (T = 0.046). The dashed line indicates the threshold (Tth = 0.2), demonstrating the NOT gate’s high CR of 11.40 dB.

Figure 11 shows the input and output transmission spectra for the NOT gate, demonstrating its operational principle of logical inversion through resonant phase-tuning in the microring at 1550 nm. Figure 11a displays the input spectra for the two logic states, while Figure 11b confirms the NOT truth table: the output state is the inverse of the input state. An input logic ‘0’ produces a high transmission peak (T = 0.635), significantly exceeding Tth = 0.2 and registering a logic ‘1’. In stark contrast, an input logic ‘1’ results in a profoundly suppressed resonance (T = 0.046), falling well below the threshold to register a definitive logic ‘0’. The consistent spectral inversion behavior showing high transmission for input ‘0’ and deep suppression for input ‘1’, both precisely centered at the operational wavelength, visually validates the gate’s high CR of 11.40 dB and its robust performance for photonic logic networks.

### 3.5. NOR

**Figure 12:**
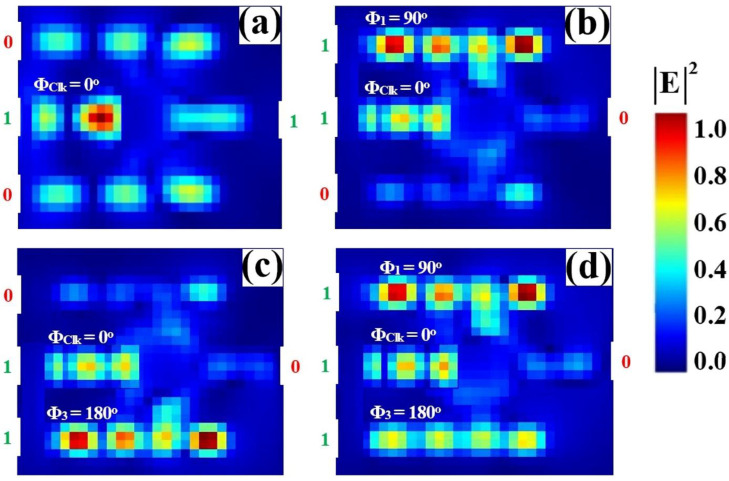
Numerical simulation results demonstrating NOR gate operation on the SoS waveguide at 1550 nm. The output transmission intensity for all four input combinations: (**a**) ‘00’, (**b**) ‘10’, (**c**) ‘01’, and (**d**) ‘11’.

The NOR logic function is implemented using the same interferometric SoS platform, operating at 1550 nm. As schematically represented in Figure 12, a Clk with a fixed phase (Φ_Clk_ = 0°) is injected into P_in2_. The two data inputs are applied to ports P_in1_ and P_in3_. The gate’s operation is defined by its output being high only when all inputs are low. When both inputs are at logic ‘0’, the unimpeded Clk signal establishes CI, yielding a high output transmission of T = 0.984, which is interpreted as a logic ‘1’. Conversely, the presence of any logic ‘1’ at the input ports, for states ‘10’, ‘01’, or ‘11’, introduces a phase mismatch that forces DI. This suppresses the output field, resulting in low transmission values of T = 0.041, 0.022, and 0.041, respectively, all corresponding to a logic ‘0’. This negative-logic operation, yielding a high output solely for the ‘00’ input condition, successfully validates the NOR truth table. The significant extinction between the single high-output state and the three low-output states confirms the design’s robustness for executing complex logic operations in all-optical computing.

Table 5 quantifies the performance metrics of the all-optical NOR gate, confirming its operation in accordance with the truth table. The output is ‘1’ only when both data inputs are ‘0’ (‘00’), resulting in a high transmission of T = 0.635. For all other input combinations where at least one data input is ‘1’—‘10’, ‘01’, and ‘11’—the output is logic ‘0’, with strongly suppressed transmissions of T = 0.035, 0.035, and 0.041, respectively. The high contrast ratio of 13.35 dB demonstrates a robust distinction between the single high-output state and the multiple low-output states, validating the gate’s reliability for cascadable photonic logic circuits.

**Figure 13:**
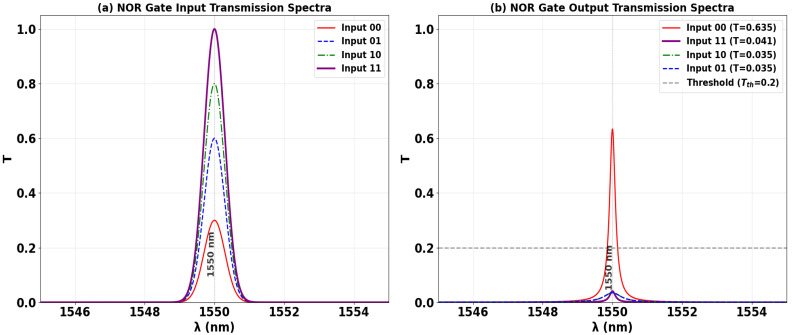
(**a**) Input transmission spectra for the four input combinations of the NOR gate at operating wavelength (λ = 1550 nm). (**b**) Output transmission spectra (T) showing high transmission for logic ‘1’ state (input ‘00’ with T = 0.635) and profound suppression for logic ‘0’ states (inputs ‘10’, ‘01’, and ‘11’ with T = 0.035, T = 0.035, and T = 0.041, respectively). The dashed line indicates the threshold (Tth = 0.2), demonstrating the NOR gate’s high CR of 13.35 dB.

Figure 13 shows the input and output transmission spectra for the NOR gate, demonstrating its operational principle through resonant phase-tuning in the microring at 1550 nm. Figure 13a displays the input spectra for the four logic combinations, while Figure 13b confirms the NOR truth table: the output is logic ‘1’ only when both inputs are ‘0’. The ‘00’ input state produces a high transmission peak (T = 0.635), significantly exceeding Tth = 0.2. In stark contrast, the input states ‘10’, ‘01’, and ‘11’ all result in profoundly suppressed resonances (T = 0.035, T = 0.035, and T = 0.041, respectively), falling well below the threshold to register definitive logic ‘0’ states. The consistent spectral behavior showing one high-transmission state and three suppressed states, all precisely centered at the operational wavelength, visually validates the gate’s high CR of 13.35 dB and its robust performance for advanced photonic logic networks.

### 3.6. NAND

**Figure 14:**
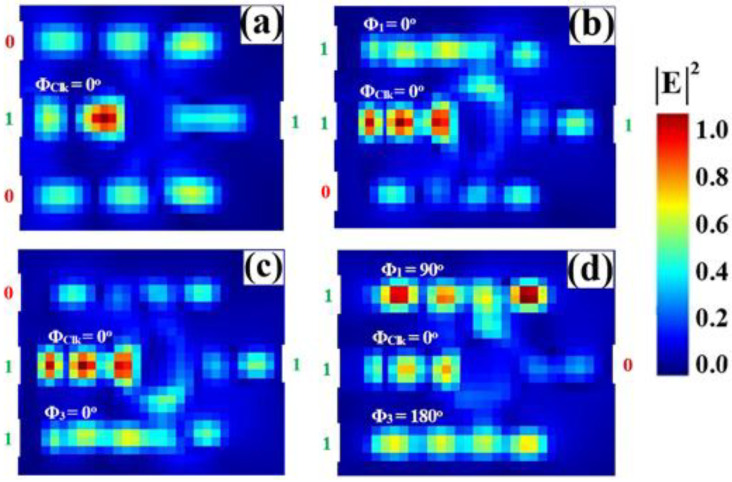
Numerical simulation results demonstrating NAND gate operation on the SoS waveguide at 1550 nm. The output transmission intensity for all four input combinations: (**a**) ‘00’, (**b**) ‘10’, (**c**) ‘01’, and (**d**) ‘11’.

The NAND logic function is implemented on the same SoS platform, completing the suite of fundamental all-optical gates. As configured in Figure 14, a Clk with a fixed phase (Φ_Clk_ = 0°) is injected into P_in2_, while the data inputs are applied to P_in1_ and P_in3_. The gate’s operation is defined by its output being low only when all inputs are high. When both inputs are at logic ‘1’, their specific phase settings (e.g., Φ_1_ = 90°, Φ_3_ = 180°) are calibrated to destructively interfere with the Clk signal. This forced phase mismatch suppresses the resonant field, yielding a low output transmission of T ≈ 0.041, which is interpreted as a logic ‘0’. Conversely, for any other input combination (‘00’, ‘01’, ‘10’), the inputs facilitate CI with the Clk. This results in high output transmission states, corresponding to logic ‘1’. This confirms the NAND truth table, where the output is ‘0’ solely for the ‘11’ input condition. The clear demarcation between the single low-output state and the multiple high-output states underscores the design’s versatility and its capability to execute complex Boolean functions.

Table 6 quantifies the performance metrics of the all-optical NAND gate, confirming its truth table operation. The output is logic ‘0’ only when both data inputs are ‘1’ (‘11’), yielding a strongly suppressed transmission of T = 0.041. For all other input combinations—‘00’, ‘10’, and ‘01’—the output is logic ‘1’, with high transmissions of T = 0.635, 0.674, and 0.674, respectively. The high CR of 12.10 dB demonstrates a robust distinction between the single low-output state and the multiple high-output states.

**Figure 15:**
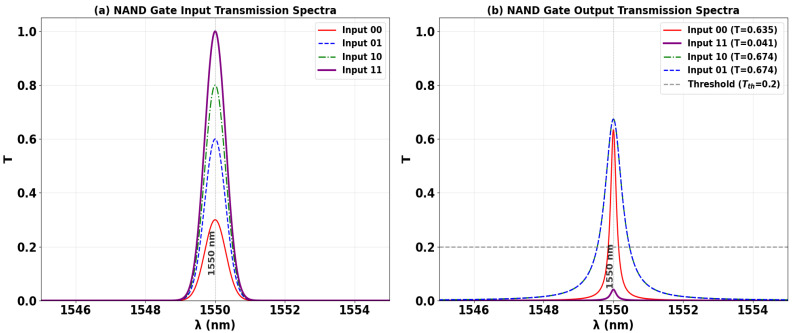
(**a**) Input transmission spectra for the four input combinations of the NAND gate at operating wavelength (λ = 1550 nm). (**b**) Output transmission spectra (T) showing high transmission for logic ‘1’ states (inputs ‘00’, ‘01’, and ‘10’ with T = 0.635, T = 0.674, and T = 0.674, respectively) and profound suppression for logic ‘0’ state (input ‘11’ with T = 0.041). The dashed line indicates the threshold (Tth = 0.2), demonstrating the NAND gate’s high CR of 12.10 dB.

Figure 15 shows the input and output transmission spectra for the NAND gate, demonstrating its operational principle through resonant phase-tuning in the microring at 1550 nm. Figure 15a displays the input spectra for the four logic combinations, while Figure 15b confirms the NAND truth table: the output is logic ‘0’ only when both inputs are ‘1’. The ‘11’ input state produces a profoundly suppressed resonance (T = 0.041), falling well below Tth = 0.2 to register a definitive logic ‘0’. In stark contrast, the input states ‘00’, ‘01’, and ‘10’ all generate distinct high transmission peaks (T = 0.635, T = 0.674, and T = 0.674, respectively), each significantly exceeding the threshold and corresponding to a logic ‘1’. The consistent spectral behavior showing three high-transmission states and one suppressed state, all precisely centered at the operational wavelength, visually validates the gate’s high CR of 12.10 dB and its robust performance for advanced photonic computing.

### 3.7. XNOR

**Figure 16:**
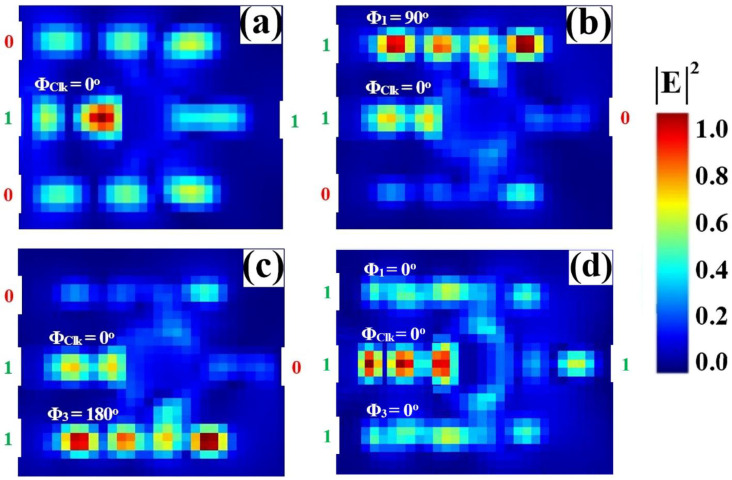
Numerical simulation results demonstrating XNOR gate operation on the SoS waveguide at 1550 nm. The output transmission intensity for all four input combinations: (**a**) ‘00’, (**b**) ‘10’, (**c**) ‘01’, and (**d**) ‘11’.

The XNOR logic function, which outputs ‘1’ when both inputs are identical, is realized on the SoS platform through precise phase coherence control. In the configuration shown in Figure 16, the two data signals are applied to ports P_in1_ and P_in3_, while a Clk with a fixed phase (Φ_Clk_ = 0°) is injected into P_in2_ to serve as a reference. The gate operation is determined by the phase relationship between the two data inputs relative to the Clk. When the inputs are phase-matched (e.g., both at 0°), they constructively interfere with the Clk signal. This resonant buildup produces a high output transmission of T ≈ 0.886, registering a logic ‘1’. Conversely, when the inputs are phase-mismatched (e.g., Φ_1_ = 90°, Φ_3_ = 180°), they destructively interfere with the Clk, quenching the cavity field. This suppression results in a low output transmission that falls below the decision threshold, yielding a logic ‘0’. This result confirms the XNOR truth table operation, where the output is high only for identical inputs (‘00’ or ‘11’) and low for divergent inputs (‘01’ or ‘10’). The ability to execute this equivalence function demonstrates the versatility of the phase-based computing paradigm for implementing complex Boolean logic in photonic integrated circuits.

Table 7 quantifies the performance metrics of the all-optical XNOR gate, confirming its operation in accordance with the equivalence logic truth table. The output is logic ‘1’ when both data inputs are identical—for input states ‘00’ (T = 0.635) and ‘11’ (T = 0.674). Conversely, the output is logic ‘0’ when the inputs differ, for states ‘01’ and ‘10’, yielding strongly suppressed transmissions of T = 0.035 for both. The high contrast ratio of 12.72 dB demonstrates a robust distinction between the dual high-output states (for matched inputs) and the dual low-output states (for divergent inputs), validating the gate’s reliable XNOR functionality.

**Figure 17:**
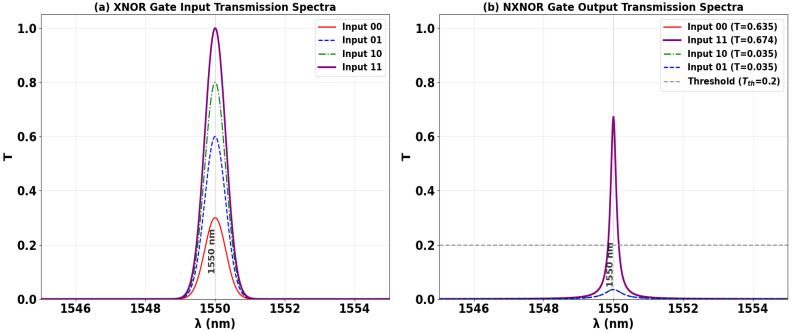
(**a**) Input transmission spectra for the four input combinations of the XNOR gate at operating wavelength (λ = 1550 nm). (**b**) Output transmission spectra (T) showing high transmission for logic ‘1’ states (inputs ‘00’ and ‘11’ with T = 0.635 and T = 0.674, respectively) and profound suppression for logic ‘0’ states (inputs ‘10’ and ‘01’ with T = 0.035 and T = 0.035, respectively). The dashed line indicates the threshold (Tth = 0.2), demonstrating the XNOR gate’s high CR of 12.72 dB.

Figure 17 shows the input and output transmission spectra for the XNOR gate, demonstrating its operational principle of logical equivalence through resonant phase-tuning in the microring at 1550 nm. Figure 17a displays the input spectra for the four logic combinations, while Figure 17b confirms the XNOR truth table: the output is logic ‘1’ only when both inputs are identical. The input states ‘00’ and ‘11’ each produce distinct high transmission peaks (T = 0.635 and T = 0.674, respectively), significantly exceeding Tth = 0.2. In stark contrast, the divergent input states ‘10’ and ‘01’ result in profoundly suppressed resonances (T = 0.035 for both), falling well below the threshold to register definitive logic ‘0’ states. The consistent spectral behavior showing two high-transmission states for identical inputs and two suppressed states for divergent inputs, all precisely centered at the operational wavelength, visually validates the gate’s high CR of 12.72 dB and its robust performance for photonic logic applications requiring equivalence checking.

## 4. Performance Comparison

Table 8 presents a detailed comparative analysis of state-of-the-art all-optical logic gate (AOLG) implementations [68,69,70,71,72,73,74,75,76,77], providing context to evaluate the contributions of the silicon-on-silica (SoS) microring resonator platform developed in this work. The results demonstrate a strategically engineered architecture that effectively balances miniaturization, operational speed, and signal fidelity. The monolithic integration of all seven fundamental Boolean logic gates (XOR, AND, OR, NOT, NOR, XNOR, NAND) is achieved with an ultra-compact footprint of 1.42 × 1.08 µm^2^ per gate, an operational bandwidth supporting data rates up to 55 Gb/s, and a consistent contrast ratio (CR) of 11.40–13.72 dB at the standard telecommunications wavelength of 1550 nm. While the achieved CR is slightly below the maximum reported in some photonic-crystal (PhC) structures (~18 dB, Ref. [73]), it remains sufficiently high for reliable high-speed logic operation.

The CR can be further improved using several optimization strategies:

- **Refractive-index contrast engineering** between core and cladding to enhance confinement and reduce unwanted losses.
- **Precise tuning of coupling gaps and cavity geometry** to sharpen resonance peaks and improve the ON/OFF intensity ratio.
- **Minimization of propagation and scattering losses** through high-quality fabrication and smooth sidewalls.
- **Advanced material engineering**, such as controlled doping or hybrid integration with low-loss dielectrics.

A comparison with existing platforms highlights key advantages of the proposed design:

1. **Ultra-compact footprint:** The active area is orders of magnitude smaller than most PhC lattices [69,70,71,72,73] and plasmonic designs [68,74,75,76], enabling dense integration in large-scale photonic circuits.
2. **CMOS compatibility and low loss:** The all-dielectric SoS platform avoids metal-induced absorption, exhibits thermal stability, and is fully compatible with mature CMOS processes, providing a cost-effective route for mass production and seamless integration with electronics.
3. **Design uniformity, modularity, and scalability:** The common microring-based resonant mechanism across all seven logic gates allows straightforward replication, cascading, and reconfiguration without individual gate redesign. This modular approach facilitates scaling to multi-gate systems and complex photonic logic networks, in contrast to PhC [69,70,71,72,73] and plasmonic designs [68,74,75,76], which often require extensive redesign for each gate.
4. **High-speed operation:** The 55 Gb/s data rate, while slightly below the theoretical maximum of certain PhC structures [69,73], significantly surpasses several experimental demonstrations (e.g., 0.31 Gb/s [74] and 20 Gb/s [75]), meeting the requirements of modern optical interconnects and computing systems.
5. **Advanced functionality:** Compared to inverse-designed ultra-compact photonic gates [77], the SoS microring platform provides a robust, easily fabricated, and modular solution suitable for large-scale integration, maintaining performance uniformity across all seven logic gates.

In summary, this work introduces a harmonious, scalable, and CMOS-compatible architecture. The combination of minimal footprint, high speed, stable signal integrity, and straightforward scalability directly addresses the key challenges of integrated photonics. The proposed SoS microring resonator platform therefore establishes a promising foundation for future densely integrated all-optical processing and computing architectures.

## 5. Conclusions

This work has comprehensively demonstrated the design, simulation, and performance of a complete suite of all-optical logic gates (AOLGs)—XOR, AND, OR, NOT, NOR, XNOR, and NAND—on a robust silicon-on-silica (SoS) waveguide platform, operating at the standard telecommunications wavelength of 1550 nm. The proposed architecture, centered around a single microring resonator coupled to multiple bus waveguides, leverages the fundamental principles of phase-mediated constructive and destructive interference to execute complex Boolean logic with high fidelity. The numerical simulation results, validated through rigorous Lumerical FDTD analysis, confirm the successful operation of all seven logic functions. The designed resonator achieved a high Q-factor of 11,071, a key indicator of low optical loss and efficient light confinement. Each gate exhibited robust performance, characterized by high output transmission for logic ‘1’ states and strong suppression for logic ‘0’ states. The calculated contrast ratios (CRs) for the gates, ranging from 11.40 dB to 13.72 dB, consistently exceed the minimum threshold required for reliable cascadability in digital optical systems. The ultra-compact footprint of 1.42 × 1.08 µm^2^ per gate underscores an exceptional degree of miniaturization, addressing one of the most critical challenges in photonic integrated circuit (PIC) design. The high refractive index contrast between the silicon core and silica cladding enabled strong optical confinement and small bending radii, while the inherent CMOS compatibility of the platform provides a practical pathway toward fabrication and eventual mass production. Benchmarking against the state-of-the-art demonstrates a balanced optimization of miniaturization, low loss, operational speed (55 Gb/s), signal integrity, and fabrication practicality. The ability to implement a universal set of logic gates within a single, monolithic architectural framework represents a significant step toward realizing fully functional, large-scale all-optical processors. For researchers and engineers adopting this architecture, several practical considerations are highlighted. Fabrication precision is critical, as performance is highly sensitive to coupling gaps, ring radii, and cavity geometries. Accurate refractive-index control is necessary to maintain resonance conditions and avoid wavelength shifts. Loss minimization through smooth waveguide sidewalls and low scattering is essential, particularly in cascaded or densely packed systems. Temperature control may be required for large-scale integration to prevent resonance shifts, and careful design of inter-gate spacing and signal routing is recommended to maintain signal integrity. Importantly, this study also emphasizes the ongoing transition from simulation to experimental validation. Future work will focus on prototype fabrication and characterization to confirm the simulated performance, assess power budgets, evaluate fabrication tolerances, and examine cascading feasibility. These efforts aim to bridge the current gap between theoretical modeling and practical implementation, reinforcing the credibility and applicability of the proposed architecture.

In conclusion, this research establishes the SoS microring resonator as a highly promising and competitive foundational technology for future optical computing. Its demonstrated performance, combined with its scalability, high-Q resonance, and precise phase control, offers a clear pathway toward both classical and quantum photonic logic operations. By validating these devices experimentally, the platform’s potential as a core building block for complex photonic circuits—such as arithmetic logic units, registers, and optical processing nodes, can be firmly established, enabling the development of next-generation communication and data processing systems.

## Figures and Tables

**Table 1:** Simulation XOR gate performance metrics: output logic state, normalized transmission (T), and contrast ratio (CR) for the proposed SoS waveguide at 1550 nm (Tth = 0.2).

Logic Gate	Input Signals			Output Logic	T	CR (dB)
	P_in1_ (Clk)	P_in2_	P_in3_	P_out_		
XOR	1	0	0	0	0.035	13.41
	1	1	0	1	0.675	
	1	0	1	1	0.925	
	1	1	1	0	0.038	

**Table 2:** Simulation AND gate performance metrics: output logic state, normalized transmission (T), and contrast ratio (CR) for the proposed SoS waveguide at 1550 nm (Tth = 0.2).

Logic Gate	Input Signals			Output Logic	T	CR (dB)
	P_in1_ (Clk)	P_in2_	P_in3_	P_out_		
AND	1	0	0	0	0.035	13.58
	1	1	0	0	0.043	
	1	0	1	0	0.037	
	1	1	1	1	0.874	

**Table 3:** Simulation OR gate performance metrics: output logic state, normalized transmission (T), and contrast ratio (CR) for the proposed SoS waveguide at 1550 nm (Tth = 0.2).

Logic Gate	Input Signals			Output Logic	T	CR (dB)
	P_in1_ (Clk)	P_in2_	P_in3_	P_out_		
OR	1	0	0	0	0.035	13.72
	1	1	0	1	0.675	
	1	0	1	1	0.925	
	1	1	1	1	0.874	

**Table 4:** Simulation NOT gate performance metrics: output logic state, normalized transmission (T), and contrast ratio (CR) for the proposed SoS waveguide at 1550 nm (Tth = 0.2).

Logic Gate	Input Signals		Output Logic	T	CR
	P_in2_ (Clk)	P_in3_	P_out_		
NOT	1	0	1	0.635	11.40
	1	1	0	0.046	

**Table 5:** Simulation NOR gate performance metrics: output logic state, normalized transmission (T), and contrast ratio (CR) for the proposed SoS waveguide at 1550 nm (Tth = 0.2).

Logic Gate	Input Signals			Output Logic	T	CR (dB)
	P_in1_	P_in2_ (Clk)	P_in3_	P_out_		
NOR	0	1	0	1	0.635	13.35
	1	1	0	0	0.035	
	0	1	1	0	0.035	
	1	1	1	0	0.041	

**Table 6:** Simulation NAND gate performance metrics: output logic state, normalized transmission (T), and contrast ratio (CR) for the proposed SoS waveguide at 1550 nm (Tth = 0.2).

Logic Gate	Input Signals			Output Logic	T	CR (dB)
	P_in1_	P_in2_ (Clk)	P_in3_	P_out_		
NAND	0	1	0	1	0.635	12.10
	1	1	0	1	0.674	
	0	1	1	1	0.674	
	1	1	1	0	0.041	

**Table 7:** Simulation XNOR gate performance metrics: output logic state, normalized transmission (T), and contrast ratio (CR) for the proposed SoS waveguide at 1550 nm (Tth = 0.2).

Logic Gate	Input Signals			Output Logic	T	CR (dB)
	P_in1_	P_in2_ (Clk)	P_in3_	P_out_		
XNOR	0	1	0	1	0.635	12.72
	1	1	0	0	0.035	
	0	1	1	0	0.035	
	1	1	1	1	0.674	

**Table 8:** Comprehensive comparative analysis of all-optical logic gate architectures. The table benchmarks key performance indicators—including footprint, operational speed (Gb/s), operating wavelength (λ), and signal quality metric (extinction ratio (ER)/contrast ratio (CR)), across diverse platforms such as silicon photonics, photonic crystals (PhC), and plasmonics. The results from this work (final row) demonstrate a competitive balance of ultra-compact size, high speed, and strong contrast ratio within a CMOS-compatible platform.

Logic Gates	Waveguide	Materials	Size (µm^2^)	Speed (Gb/s)	λ (nm)	Metric (dB)	Exp./Sim.	Ref.
AND, NAND	Silicon micro-ring resonators	Si/SiO_2_	5 µm radius	0.310	1550.7	ER ~ 10	Exp.	[27]
XOR, AND, OR, NOT, NOR, XNOR, NAND	Silicon microrings waveguide	Si/SiO_2_	1.30 × 1.35	199.80	1550	CR = 12.02–15.85	Sim.	[50]
AND, NOR, XNOR	Si photonics platform	Si	3 µm long	20	1550	CR > 10	Exp.	[67]
NOT, OR, AND, NOR, NAND, XOR, XNOR	Dielectric-metal-dielectric plasmonic waveguide	Silver/Teflon	-	-	900–1330	ER > 20	Sim.	[68]
AND, XOR, OR, NOT, NAND, NOR, XNOR	PhC waveguides	Si/Air	5.28 × 5.28	976	1550	CR = 5.42–9.59	Sim.	[69]
AND, XOR, XNOR	T-shaped PhC waveguides	Si/Air	8.4 × 5.4	>30,000	1550	CR = 8.29–33.05	Sim.	[70,71,72]
AND, OR	2D PhC design	Si/Air	19.8 × 12.6	>4740	1520	CR = 9.74 and 17.95	Sim.	[73]
NOT, XOR, AND, OR, NOR, NAND, XNOR	Metal slot waveguide	Silver/SiO_2_	1.5 × 2.36	-	632.8	CR = 6–16	Exp.	[74]
NOT, XOR, AND, OR, NOR, NAND, XNOR	Metal-insulator-metal structures	Air/Silver	5.33 × 0.42	-	632.8	CR = 15	Sim.	[75]
AND, NAND, OR, XOR, NOR, XNOR, NOT	Plasmonic logic gate design	Silver/SiO_2_	0.25 × 0.25	-	850	CR = 4.14–14.46	Sim.	[76]
AND, OR, NOT, NAND	Inverse design on silicon platforms	Si/SiO_2_	1.0 × 1.5	-	1300	CR = 0.5–5.79	Sim.	[77]
XOR, AND, OR, NOT, NOR, XNOR, NAND	Silicon racetrack and ring resonator	Si/SiO_2_	1.42 × 1.08	55	1550	CR = 11.40–13.72	Sim.	This work

## Data Availability

Data are contained within the article.

## List of Abbreviations

FDTD	finite-difference time-domain
AOLGs	all-optical logic gates
CR	contrast ratio
Si	silicon
SiO_2_	silicon dioxide
CMOS	complementary metal-oxide semiconductor
T	normalized transmission
Q	quality factor
TE	transverse electric mode
b	ring outer radius
a	ring inner radius
w	ring width
g	coupling gap
Clk	clock signal
CI	constructive interference
DI	destructive interference
τₚ	photon lifetime
λ	operating wavelength
FWHM	full width at half maximum
